# Supplementary material for: HIVprotI: an integrated web based platform for prediction and design of HIV proteins inhibitors
Source: J Cheminform. 2018 Mar 9;10:12. doi: 10.1186/s13321-018-0266-y (PMC5845081; doi:10.1186/s13321-018-0266-y)
Supplement: Supplementary file 2 — Additional file 2. Source code of HIVProtI web server. [file 13321_2018_266_MOESM2_ESM.zip › HIVprotI_Source-code/hivprotI/weka-3-6-6/doc/weka/gui/beans/Saver.html]

Saver


---


|  |  |  |
| --- | --- | --- |
| **Overview** \| **Package** \| **Class** \| **Tree** \| **Deprecated** \| **Index** \| **Help** \| **Weka's home** | |  |
| **PREV CLASS**   **NEXT CLASS** | **FRAMES**    **NO FRAMES**     **All Classes** |  |
| SUMMARY: NESTED \| FIELD \| CONSTR \| METHOD | DETAIL: FIELD \| CONSTR \| METHOD |  |


---


## weka.gui.beans Class Saver

```
java.lang.Object
  java.awt.Component
      java.awt.Container
          javax.swing.JComponent
              javax.swing.JPanel
                  weka.gui.beans.AbstractDataSink
                      weka.gui.beans.Saver
```

**All Implemented Interfaces:**: java.awt.image.ImageObserver, java.awt.MenuContainer, java.io.Serializable, java.util.EventListener, javax.accessibility.Accessible, EnvironmentHandler, BeanCommon, DataSink, DataSourceListener, InstanceListener, TestSetListener, ThresholdDataListener, TrainingSetListener, Visible, WekaWrapper

---

``` public class Saver extends AbstractDataSink implements WekaWrapper, EnvironmentHandler ```

Saves data sets using weka.core.converter classes

**Version:**
:   $Revision: 7710 $

**Author:**
:   Stefan Mutter

**See Also:**: Serialized Form

---

**Nested Class Summary**

**Nested classes/interfaces inherited from class javax.swing.JComponent**

`javax.swing.JComponent.AccessibleJComponent`

**Nested classes/interfaces inherited from class java.awt.Component**

`java.awt.Component.BaselineResizeBehavior`


**Field Summary**

**Fields inherited from class javax.swing.JComponent**

`TOOL_TIP_TEXT_KEY, UNDEFINED_CONDITION, WHEN_ANCESTOR_OF_FOCUSED_COMPONENT, WHEN_FOCUSED, WHEN_IN_FOCUSED_WINDOW`

**Fields inherited from class java.awt.Component**

`BOTTOM_ALIGNMENT, CENTER_ALIGNMENT, LEFT_ALIGNMENT, RIGHT_ALIGNMENT, TOP_ALIGNMENT`

**Fields inherited from interface java.awt.image.ImageObserver**

`ABORT, ALLBITS, ERROR, FRAMEBITS, HEIGHT, PROPERTIES, SOMEBITS, WIDTH`


**Constructor Summary**

`Saver()`
  
          Contsructor


| **Method Summary** | |
| --- | --- |
| `void` | `acceptDataSet(DataSetEvent e)`             Method reacts to a dataset event and starts the writing process in batch mode |
| `void` | `acceptDataSet(ThresholdDataEvent e)`             Method reacts to a threshold data event ans starts the writing process in batch mode. |
| `void` | `acceptInstance(InstanceEvent e)`             Methods reacts to instance events and saves instances incrementally. |
| `void` | `acceptTestSet(TestSetEvent e)`             Method reacts to a test set event and starts the writing process in batch mode |
| `void` | `acceptTrainingSet(TrainingSetEvent e)`             Method reacts to a training set event and starts the writing process in batch mode |
| `java.lang.String` | `getCustomName()`             Get the custom (descriptive) name for this bean (if one has been set) |
| `boolean` | `getRelationNameForFilename()`             Get whether the relation name is the primary part of the filename. |
| `Saver` | `getSaverTemplate()`             Get the saver |
| `java.lang.Object` | `getWrappedAlgorithm()`             Get the saver |
| `java.lang.String` | `globalInfo()`             Global info (if it exists) for the wrapped loader |
| `boolean` | `isBusy()`             Returns true if. |
| `static void` | `main(java.lang.String[] args)`             The main method for testing |
| `void` | `saveBatch()`             Saves instances in batch mode |
| `void` | `setCustomName(java.lang.String name)`             Set a custom (descriptive) name for this bean |
| `void` | `setEnvironment(Environment env)`             Set environment variables to use. |
| `void` | `setRelationNameForFilename(boolean r)`             Set whether to use the relation name as the primary part of the filename. |
| `void` | `setSaverTemplate(Saver saver)`             Set the loader to use |
| `void` | `setWrappedAlgorithm(java.lang.Object algorithm)`             Set the saver |
| `void` | `stop()`             Stops the bean |

**Methods inherited from class weka.gui.beans.AbstractDataSink**

`connectionAllowed, connectionAllowed, connectionNotification, disconnectionNotification, getVisual, setLog, setVisual, useDefaultVisual`

**Methods inherited from class javax.swing.JPanel**

`getAccessibleContext, getUI, getUIClassID, setUI, updateUI`

**Methods inherited from class javax.swing.JComponent**

`addAncestorListener, addNotify, addVetoableChangeListener, computeVisibleRect, contains, createToolTip, disable, enable, firePropertyChange, firePropertyChange, firePropertyChange, getActionForKeyStroke, getActionMap, getAlignmentX, getAlignmentY, getAncestorListeners, getAutoscrolls, getBaseline, getBaselineResizeBehavior, getBorder, getBounds, getClientProperty, getComponentPopupMenu, getConditionForKeyStroke, getDebugGraphicsOptions, getDefaultLocale, getFontMetrics, getGraphics, getHeight, getInheritsPopupMenu, getInputMap, getInputMap, getInputVerifier, getInsets, getInsets, getListeners, getLocation, getMaximumSize, getMinimumSize, getNextFocusableComponent, getPopupLocation, getPreferredSize, getRegisteredKeyStrokes, getRootPane, getSize, getToolTipLocation, getToolTipText, getToolTipText, getTopLevelAncestor, getTransferHandler, getVerifyInputWhenFocusTarget, getVetoableChangeListeners, getVisibleRect, getWidth, getX, getY, grabFocus, isDoubleBuffered, isLightweightComponent, isManagingFocus, isOpaque, isOptimizedDrawingEnabled, isPaintingForPrint, isPaintingTile, isRequestFocusEnabled, isValidateRoot, paint, paintImmediately, paintImmediately, print, printAll, putClientProperty, registerKeyboardAction, registerKeyboardAction, removeAncestorListener, removeNotify, removeVetoableChangeListener, repaint, repaint, requestDefaultFocus, requestFocus, requestFocus, requestFocusInWindow, resetKeyboardActions, reshape, revalidate, scrollRectToVisible, setActionMap, setAlignmentX, setAlignmentY, setAutoscrolls, setBackground, setBorder, setComponentPopupMenu, setDebugGraphicsOptions, setDefaultLocale, setDoubleBuffered, setEnabled, setFocusTraversalKeys, setFont, setForeground, setInheritsPopupMenu, setInputMap, setInputVerifier, setMaximumSize, setMinimumSize, setNextFocusableComponent, setOpaque, setPreferredSize, setRequestFocusEnabled, setToolTipText, setTransferHandler, setVerifyInputWhenFocusTarget, setVisible, unregisterKeyboardAction, update`

**Methods inherited from class java.awt.Container**

`add, add, add, add, add, addContainerListener, addPropertyChangeListener, addPropertyChangeListener, applyComponentOrientation, areFocusTraversalKeysSet, countComponents, deliverEvent, doLayout, findComponentAt, findComponentAt, getComponent, getComponentAt, getComponentAt, getComponentCount, getComponents, getComponentZOrder, getContainerListeners, getFocusTraversalKeys, getFocusTraversalPolicy, getLayout, getMousePosition, insets, invalidate, isAncestorOf, isFocusCycleRoot, isFocusCycleRoot, isFocusTraversalPolicyProvider, isFocusTraversalPolicySet, layout, list, list, locate, minimumSize, paintComponents, preferredSize, printComponents, remove, remove, removeAll, removeContainerListener, setComponentZOrder, setFocusCycleRoot, setFocusTraversalPolicy, setFocusTraversalPolicyProvider, setLayout, transferFocusBackward, transferFocusDownCycle, validate`

**Methods inherited from class java.awt.Component**

`action, add, addComponentListener, addFocusListener, addHierarchyBoundsListener, addHierarchyListener, addInputMethodListener, addKeyListener, addMouseListener, addMouseMotionListener, addMouseWheelListener, bounds, checkImage, checkImage, contains, createImage, createImage, createVolatileImage, createVolatileImage, dispatchEvent, enable, enableInputMethods, firePropertyChange, firePropertyChange, firePropertyChange, firePropertyChange, firePropertyChange, getBackground, getBounds, getColorModel, getComponentListeners, getComponentOrientation, getCursor, getDropTarget, getFocusCycleRootAncestor, getFocusListeners, getFocusTraversalKeysEnabled, getFont, getForeground, getGraphicsConfiguration, getHierarchyBoundsListeners, getHierarchyListeners, getIgnoreRepaint, getInputContext, getInputMethodListeners, getInputMethodRequests, getKeyListeners, getLocale, getLocation, getLocationOnScreen, getMouseListeners, getMouseMotionListeners, getMousePosition, getMouseWheelListeners, getName, getParent, getPeer, getPropertyChangeListeners, getPropertyChangeListeners, getSize, getToolkit, getTreeLock, gotFocus, handleEvent, hasFocus, hide, imageUpdate, inside, isBackgroundSet, isCursorSet, isDisplayable, isEnabled, isFocusable, isFocusOwner, isFocusTraversable, isFontSet, isForegroundSet, isLightweight, isMaximumSizeSet, isMinimumSizeSet, isPreferredSizeSet, isShowing, isValid, isVisible, keyDown, keyUp, list, list, list, location, lostFocus, mouseDown, mouseDrag, mouseEnter, mouseExit, mouseMove, mouseUp, move, nextFocus, paintAll, postEvent, prepareImage, prepareImage, remove, removeComponentListener, removeFocusListener, removeHierarchyBoundsListener, removeHierarchyListener, removeInputMethodListener, removeKeyListener, removeMouseListener, removeMouseMotionListener, removeMouseWheelListener, removePropertyChangeListener, removePropertyChangeListener, repaint, repaint, repaint, resize, resize, setBounds, setBounds, setComponentOrientation, setCursor, setDropTarget, setFocusable, setFocusTraversalKeysEnabled, setIgnoreRepaint, setLocale, setLocation, setLocation, setName, setSize, setSize, show, show, size, toString, transferFocus, transferFocusUpCycle`

**Methods inherited from class java.lang.Object**

`equals, getClass, hashCode, notify, notifyAll, wait, wait, wait`

**Constructor Detail**

### Saver

```
public Saver()
```

:   Contsructor


**Method Detail**

### isBusy

```
public boolean isBusy()
```

:   Returns true if. at this time, the bean is busy with some
    (i.e. perhaps a worker thread is performing some calculation).

    :   **Specified by:**: `isBusy` in interface `BeanCommon`
    :   **Returns:**: true if the bean is busy.

---


### globalInfo

```
public java.lang.String globalInfo()
```

:   Global info (if it exists) for the wrapped loader

    :   **Returns:**: the global info

---


### setCustomName

```
public void setCustomName(java.lang.String name)
```

:   Set a custom (descriptive) name for this bean

    :   **Specified by:**: `setCustomName` in interface `BeanCommon`
    :   **Parameters:**: `name` - the name to use

---


### getCustomName

```
public java.lang.String getCustomName()
```

:   Get the custom (descriptive) name for this bean (if one has been set)

    :   **Specified by:**: `getCustomName` in interface `BeanCommon`
    :   **Returns:**: the custom name (or the default name)

---


### setEnvironment

```
public void setEnvironment(Environment env)
```

:   Set environment variables to use.

    :   **Specified by:**: `setEnvironment` in interface `EnvironmentHandler`
    :   **Parameters:**: `env` - the environment variables to use

---


### setSaverTemplate

```
public void setSaverTemplate(Saver saver)
```

:   Set the loader to use

    :   **Parameters:**: `saver` - a Saver

---


### acceptDataSet

```
public void acceptDataSet(DataSetEvent e)
```

:   Method reacts to a dataset event and starts the writing process in batch mode

    :   **Specified by:**: `acceptDataSet` in interface `DataSourceListener` **Specified by:**: `acceptDataSet` in class `AbstractDataSink`
    :   **Parameters:**: `e` - a dataset event

---


### acceptDataSet

```
public void acceptDataSet(ThresholdDataEvent e)
```

:   Method reacts to a threshold data event ans starts the writing process
    in batch mode.

    :   **Specified by:**: `acceptDataSet` in interface `ThresholdDataListener` **Specified by:**: `acceptDataSet` in class `AbstractDataSink`
    :   **Parameters:**: `e` - threshold data event.

---


### acceptTestSet

```
public void acceptTestSet(TestSetEvent e)
```

:   Method reacts to a test set event and starts the writing process in batch mode

    :   **Specified by:**: `acceptTestSet` in interface `TestSetListener` **Specified by:**: `acceptTestSet` in class `AbstractDataSink`
    :   **Parameters:**: `e` - test set event

---


### acceptTrainingSet

```
public void acceptTrainingSet(TrainingSetEvent e)
```

:   Method reacts to a training set event and starts the writing process in batch
    mode

    :   **Specified by:**: `acceptTrainingSet` in interface `TrainingSetListener` **Specified by:**: `acceptTrainingSet` in class `AbstractDataSink`
    :   **Parameters:**: `e` - a training set event

---


### saveBatch

```
public void saveBatch()
```

:   Saves instances in batch mode

---


### acceptInstance

```
public void acceptInstance(InstanceEvent e)
```

:   Methods reacts to instance events and saves instances incrementally.
    If the instance to save is null, the file is closed and the saving process is
    ended.

    :   **Specified by:**: `acceptInstance` in interface `InstanceListener` **Specified by:**: `acceptInstance` in class `AbstractDataSink`
    :   **Parameters:**: `e` - instance event

---


### getSaverTemplate

```
public Saver getSaverTemplate()
```

:   Get the saver

    :   **Returns:**: a `weka.core.converters.Saver` value

---


### setWrappedAlgorithm

```
public void setWrappedAlgorithm(java.lang.Object algorithm)
```

:   Set the saver

    :   **Specified by:**: `setWrappedAlgorithm` in interface `WekaWrapper`
    :   **Parameters:**: `algorithm` - a Saver

---


### getWrappedAlgorithm

```
public java.lang.Object getWrappedAlgorithm()
```

:   Get the saver

    :   **Specified by:**: `getWrappedAlgorithm` in interface `WekaWrapper`
    :   **Returns:**: a Saver

---


### setRelationNameForFilename

```
public void setRelationNameForFilename(boolean r)
```

:   Set whether to use the relation name as the primary part
    of the filename. If false, then the prefix becomes the filename.

    :   **Parameters:**: `r` - true if the relation name is to be part of the filename.

---


### getRelationNameForFilename

```
public boolean getRelationNameForFilename()
```

:   Get whether the relation name is the primary part of the filename.

    :   **Returns:**: true if the relation name is part of the filename.

---


### stop

```
public void stop()
```

:   Stops the bean

    :   **Specified by:**: `stop` in interface `BeanCommon` **Specified by:**: `stop` in class `AbstractDataSink`

---


### main

```
public static void main(java.lang.String[] args)
```

:   The main method for testing

    :   **Parameters:**: `args` -


---


|  |  |  |
| --- | --- | --- |
| **Overview** \| **Package** \| **Class** \| **Tree** \| **Deprecated** \| **Index** \| **Help** \| **Weka's home** | |  |
| **PREV CLASS**   **NEXT CLASS** | **FRAMES**    **NO FRAMES**     **All Classes** |  |
| SUMMARY: NESTED \| FIELD \| CONSTR \| METHOD | DETAIL: FIELD \| CONSTR \| METHOD |  |


---
